# Hormonal Induction and Roles of Disabled-2 in Lactation and Involution

**DOI:** 10.1371/journal.pone.0110737

**Published:** 2014-10-31

**Authors:** Wensi Tao, Robert Moore, Elizabeth R. Smith, Xiang-Xi Xu

**Affiliations:** Sylvester Comprehensive Cancer Center, and Department of Cell Biology, Graduate Program in Molecular Cell and Developmental Biology, University of Miami Miller School of Medicine, Miami, Florida, United States of America; Institute of Biomedicine, Finland

## Abstract

Disabled-2 (Dab2) is a widely expressed endocytic adaptor that was first isolated as a 96 KDa phospho-protein, p96, involved in MAPK signal transduction. Dab2 expression is lost in several cancer types including breast cancer, and Dab2 is thought to have a tumor suppressor function. In mammary epithelia, Dab2 was induced upon pregnancy and further elevated during lactation. We constructed mutant mice with a mosaic Dab2 gene deletion to bypass early embryonic lethality and to investigate the roles of Dab2 in mammary physiology. Loss of Dab2 had subtle effects on lactation, but Dab2-deficient mammary glands showed a strikingly delayed cell clearance during involution. In primary cultures of mouse mammary epithelial cells, Dab2 proteins were also induced by estrogen, progesterone, and/or prolactin. Dab2 null mammary epithelial cells were refractory to growth suppression induced by TGF-beta. However, Dab2 deletion did not affect Smad2 phosphorylation; rather TGF-beta-stimulated MAPK activation was enhanced in Dab2-deficient cells. We conclude that Dab2 expression is induced by hormones and Dab2 plays a role in modulating TGF-beta signaling to enhance apoptotic clearance of mammary epithelial cells during involution.

## Introduction

Disabled-2 (Dab2), a mammalian ortholog of *Drosophila* Disabled [1], was first isolated from a murine macrophage cell line as a phospho-protein, p96, involved in CSF-1 signal transduction [2]. Dab2 is widely expressed [3], [4], but another ortholog, Dab1, is restricted to the brain [5]. The Dab2 gene produces several spliced isoforms, and p96 and p67 are the major species [2]. A Dab2 cDNA fragment isolated by a differential expression screen was referred to as DOC-2 (differentially expressed in ovarian cancer), and Dab2 mRNA was found lost in ovarian cancer [6]. Additional experiments further substantiated Dab2 to be a tumor suppressor in ovarian cancer [4], [7]. Moreover, Dab2 was identified as a down regulated gene in carcinogen-induced mammary tumors in rodents, providing the first link between Dab2 and breast cancer [8]. Several subsequent studies confirmed a reduced Dab2 expression in human breast cancer [9], [10].

Immunohistochemical staining has shown that loss of Dab2 expression occurs in 85–95% of breast and ovarian tumors, and is an early event in ovarian tumorigenicity [4]. Loss of or reduced Dab2 expression levels were also reported in many other epithelial cancer types, including colon, prostate, and head and neck. Dab2 exerts its role in directional endocytic transport and epithelial organization [11], [12], and transfection of Dab2 into ovarian and breast cancer cells lacking Dab2 expression restored the requirement of adhesion to basement membranes [10]. Thus, loss or reduction of Dab2 expression may lead to the anchorage-independent proliferation of mammary and ovarian cancer cells [10].

The domain structure of Dab2 indicates its function as an endocytic adaptor protein [13]. The N-terminus of Dab2 contains a PTB (PhosphoTyrosine Binding) domain that can bind an NPXY motif found in a subset of cell surface receptors [14]. Dab2 proteins also contain clathrin binding, NPF, and DPF motifs, which bind components of endocytic vesicles such as clathrin, AP-2, and EPS-15, respectively [15]. The C-terminus binds to the myosin VI motor protein [16], [17]. Thus, Dab2 mediates the attachment of clathrin-coated cargos containing transmembrane proteins with an NPXY motif, such as the LDL receptor, megalin, EGF receptor, and integrins, to the myosin motor, enabling their endocytosis and directional trafficking [17], [18]. A role of Dab2 in integrin trafficking and cell motility was also described [19]–[21]. Dab2 also mediates the trafficking of E-cadherin and thus epithelial organization [12]. The polarized trafficking of cell adhesion molecules such as integrins and E-cadherin may explain the role of Dab2 in epithelial polarity and organization [12]; and trafficking of surface receptors and signaling components may account for its activities in the regulation of multiple signaling pathways, including Ras/MAPK [3], [22], [23], the transforming growth factor beta (TGF-beta) [24]–[27], and Wnt [28]–[31].

Dab2 knockout results in an early embryonic lethality in mice [11], [12], [32], and the phenotype indicates that Dab2 functions in the organization of the extra-embryonic endoderm epithelium. Studies of the *dab2*-deficient embryos, embryoid bodies, and cultured cells support that Dab2 is critical for the surface targeting of cell adhesion molecules and the maintenance of epithelial polarity. Thus, Dab2 functions in endocytic trafficking to sustain cell polarity and epithelial organization, and hence loss of Dab2 leads to epithelial disorganization [12].

In addition to its role in epithelial organization, Dab2 also modulates several signaling pathways. Dab2 has been reported to function as a link between TGF-beta receptors and the Smad family proteins, aiding in the transmission of TGF-beta signaling [25]. Additional studies suggested detailed mechanisms for the participation of Dab2 in regulating TGF-beta signaling [24], [26], [27]. One study found that Dab2 loss in head and neck cancer compromised the tumor suppressor function of TGF-beta, while enabling its tumor-promoting activities, and concluded that Dab2 is a molecular switch for TGF-beta from a tumor suppressor to a promoter [24]. Dab2 contains a C-terminal proline-rich domain with sequences resembling the proline-rich domain in Sos, a guanine nucleotide exchange factor for Ras [3]. Sos binds strongly to Grb2, an adaptor linking Sos with Ras. Notably, both Sos and Dab2 bind competitively to the two SH3 domains of Grb2 via their proline-rich motifs. Thus, by competing with Sos for binding to Grb2, Dab2 can reduce the association between Sos and Grb2 and act as a negative regulator for Ras/MAPK pathway [3], [23]. Earlier studies have established that the association between Sos and Grb2 is a site of Ras/MAPK feedback regulation [33]–[38]: upon growth factor (such as insulin and EGF) stimulation, the activated MAPK also phosphorylates Sos and promotes the disassociation of Grb2 and disruption of the signaling complex [35]–[38].

In this study, we found that Dab2 expression is induced in mammary glands during lactation, and we have studied mammary glands in Dab2-deficient mice. The mammary gland is composed of a number of different cell types: epithelial cells, adipocytes, vascular endothelial cells, and stromal cells that include fibroblasts and a variety of immune cells [39]. The luminal epithelium forms the ducts and the secretory alveoli, which are embedded within the fatty stroma. A small number of reproductive hormones, including estrogen, progesterone, placental lactogen, prolactin, and oxytocin, regulate mammary development and function [40], [41], and are also implicated in breast cancer [42], [43].

The development of mammary glands has three main stages: embryonic, pubertal, and adult [44], [45]. After birth, mammary growth is arrested until puberty, when extensive elongation of the ducts accompanied by secondary branching takes place. During pregnancy, luminal epithelia proliferate rapidly and branch extensively to produce alveolar buds. A lactogenic switch occurs during late pregnancy leading to the vast production of milk proteins and lipid droplets to nourish the offspring. Finally, following the termination of lactation, mammary regression is achieved by apoptotic cell death resulting in the removal of alveolar epithelial cells, a process known as involution. During involution, around 80% of the epithelia are eliminated within a few days [45]–[47]. Mammary involution is a multiple step process, and the crucial roles of TGF-beta pathway and Bcl-2 family proteins have been studied, although the molecular aspects of signaling and regulation remain to be understood further [48]–[55].

Though the cellular mechanisms of Dab2 in endocytosis and signaling have been well studied, the in vivo relevance and relative physiological impacts of these mechanisms have not been established. In the current study, we investigated the expression and functions of Dab2 in mammary glands using Dab2 mosaic knockout mice. We also studied the mechanisms and impact of Dab2 on cellular signaling using primary mammary epithelial cells in culture.

## Materials and Methods

### Mice strains, husbandry and breeding

All experiments using lab mice have been reviewed and approved by institutional animal care and use committee (IACUC) of the University of Miami.

A new line of floxed *dab2* mice was used throughout this study, which was constructed to delete both exons 3 and 4 to avoid the production of truncated proteins from the targeted allele [56]. Here, the floxed allele is noted as (+/f) for heterozygous, (f/f) for homozygous, and (df) as deleted allele (delta flox). Previously *dab2* (f/f) mice have been characterized and the line was indistinguishable from wildtypes in the absence of Cre. Female *dab2* (f/f) and male *dab2* (+/df):Sox2-Cre (and *dab2* (+/df):Meox2-Cre) mice were used as breeding pairs. The resulting mosaics, *dab2* knockouts (*dab2* (f/df):Meox2-Cre and *dab2* (f/df):Sox2-Cre) were used as conditional knockouts, while *dab2* heterozygous (*dab2* (+/df):Meox2-Cre and *dab2* (+/df):Sox2-Cre) and floxed (*dab2* (+/f)) mice were designated as controls. The heterozygous *dab2* mice showed no detectable phenotypes and were deemed as suitable to be used as controls for the conditional knockout mice.

Meox2-Cre mice (B6.129S4-Meox2tm1(cre)Sor/J) [57] and Sox2-Cre mice (Tg(Sox2-Cre)#Amc/J) [58] were purchased from Jackson Laboratories. Mouse colonies were housed inside the barrier area of the mouse facility of University of Miami Miller School of Medicine and PCR genotyping was performed as previously described [56].

Lactating female mice were always housed individually and their litters equalized to 6 pups. To induce mammary involution the pups were removed from the female mouse after 12 days of lactation (now termed day zero of involution). For timed matings, the morning when a plug was detected was designated E 0.5. The mice were euthanized using CO_2_ (and a isoflurane vaporizer as an alternative method) inhalation for 2 min, and cervical dislocation followed to ensure the complete euthanasia of the mice before dissection and tissue collection.

### Milk harvest

Milk was harvested from postpartum mice at day 5 of lactation. The nursing mothers were separated from the pups for 12 hours before collection. To facilitate the ejection of milk, 0.5 IU of oxytocin (Sigma-Aldrich) was injected intraperitoneally. Milk was collected with gentle suction using a syringe without needle. After milk was collected, a 1∶1 volume of 2X SDS sample buffer was added. The samples were first heated on a 95°C thermoblock for 15 min and then were subjected to SDS-PAGE and Western blotting analysis.

### Whole mount mammary gland preparations

The 4^th^ inguinal mammary gland was removed at necropsy and mounted flat on glass slides. The tissues were fixed for 2 hours at 4°C with 4% paraformaldehyde in PBS and then washed extensively. The glands were stained by immersion in carmine alum solution overnight. The samples were then dehydrated in a graded ethanol series, cleared in xylene, and stored in methyl salicylate solution.

### Primary cultures of mouse mammary epithelial cells

Mammary glands were harvested at E16.5 pregnancy and cells were prepared using a modified protocol from the Bissell lab [59]. Briefly, the glands were dissected to remove fat tissues, and minced into small pieces with scissors. Cells were released by incubating the minced mammary tissues with 0.2% collagenase for 4 hours at 37°C. Organoids (epithelial cell aggregates) were collected by a brief spin in a centrifuge (5 seconds) at 1,500 rpm, which was stopped by applying the brake. The supernatant that contained mostly fibroblasts as dispersed cells was discarded. The spin and stop procedure was repeated 10 times to wash the epithelial organoids and remove fibroblasts. The epithelial organoids were placed on collagen-coated dishes to produce a culture of dispersed mammary epithelial cells. Cells were cultured in phenol red-free IMEM containing 5% charcoal-stripped FCS, ITS media supplement (Gemini Biologicals), and EGF (5 ng/ml) for 2 days before using in experiments. The resulting cells were determined to be more than 90% epithelial by immunostaining with cytokeratin-8. The cells were also positive for estrogen and progesterone receptors as determined by immunofluorescence microscopy.

For induction of Dab2 expression, estrogen (1 nM, 17 beta-estradiol), prolactin (50 ng/ml), and progesterone (1 µM) were added to cells separately or in combination. After 2–4 days, cells were harvested and analyzed by Western blot.

### Treatment of cells with TGF-beta

Recombinant mouse TGF-beta 1 was purchased from R&D Systems (7666-MB-005). Recombinant protein powder was resuspended in 1% BSA in PBS. Prior to use in experiments, the latent TGF-beta was activated by acid treatment according to the manufacturer's protocol. Dosages of TGF-beta were titrated for cell growth suppression and an optimized concentration of 10 ng/ml was used to treat mammary epithelial cells.

### Cell growth assay

Cell growth assays were performed using the cell proliferation reagent WST-1 (Roche Cat. No. 11 644 807 001). Cells were seeded at a density of 1,000 cells/well in 96-well plates in 100 µl of media. WST-1 reagent (10 µl) was added to each well in the growth media and incubated at 37°C for 1 hour. Subsequently, a colorimetric assay was performed with a scanning multi-well spectrophotometer at 460 nm to determine relative cell number. The assay was performed in triplicate.

### Protein analysis of mammary tissue by Western blot

Lysates from cells or tissues were used for Western blot analysis. Mammary epithelial cells were lysed in a 6-well dish in 0.5 ml of NP-40 IP buffer containing protease inhibitors and phosphatase inhibitors. SDS-PAGE was performed in Novex mini gel system with pre-cast 4-12% cells. The cell lysate was assayed for total protein concentration and equal protein from each sample was loaded into each lane. Proteins were then transferred onto nitrocellulose membrane. The membranes were examined for protein amount and transfer efficiency by staining with Ponceau S dye. The relative protein amount loaded to each lane was also verified by assaying for beta-actin. After blocking with 5% non-fat milk for 1 hour, membranes were incubated with primary antibodies overnight. Primary antibodies and their dilution used include: anti-Dab2 (1/5,000) (BD Transduction Laboratories, 610465), anti-Beta-actin (1/5,000) (BD Transduction Laboratories, 612657), anti-Sos1 (1/1,000) (BD Transduction Laboratories, 610096), anti-Grb2 (1/2,000) (BD Transduction Laboratories, 610112 and Santa Cruz, sc-255), anti-E-cadherin (1/2,000) (BD Transduction Laboratories, 610405), anti-N-cadherin (1/2,000) (BD Transduction Laboratories, 610921), anti-Erk1/2 (1/5,000) (BD Transduction Laboratories, 610408), anti-phospho-Erk1/2 (1/1,000) (Cell Signaling, 4370), anti-Bcl-2 (1/2,000) (Cell Signaling, 3498), anti-Bcl-xl (1/1,000) (Cell Signaling, 2764), anti-cleaved caspase-3 (1/1,000) (Cell Signaling, 9664), anti-phospho-Smad2 (1/1,000) (Cell Signaling, 9510), anti-GC-globulin (1/2,000) (Abcam, ab65636), anti-F4/80 (1/1,000) (Abcam, 6640), anti-PCNA (1/2,000) (Santa Cruz, sc-56), and anti-Beta-casein (1/2,000) (Santa Cruz, sc-30041). The secondary antibodies were conjugated with horseradish peroxidase (HRP) and were used (1/5,000 dilution) following the instructions from the manufacturer (rabbit and mouse secondary antibodies from BioRad; goat secondary antibodies from Jackson ImmunoResearch). SuperSignal West Extended Duration Substrate (Pierce Biotech) was used for chemoluminescence detection of proteins.

### Co-immunoprecipitation

Mammary epithelial cells at 80% confluency in a 6-well dish were lysed with 0.5 ml of cold NP-40 IP buffer (1% NP-40, 150 mM NaCl, 50 mM Tris-HCl, 10 mM EDTA, pH 8.0) supplemented with protease inhibitors and phosphatase inhibitors (Pierce Biotech). Cell lysates were centrifuged at 14,000 rpm for 20 min at 4°C to remove the nuclear fraction. The supernatant was incubated with specific antibodies (10 µl of anti-Grb2 per 1 ml of cell lysate) for 3 hours at 4°C. Immunoprecipitation was performed with Dynabeads protein G immunoprecipitation kit (Invitrogen). Protein G Dynabeads were added, and the mixtures were incubated for 1 hour. The beads were then collected by brief centrifugation and washed three times in IP buffer. Proteins bound to the beads were eluted in SDS-sample buffer and subjected to Western blot analysis.

### Immunohistochemistry and immunofluorescence microscopy

Tissues were fixed with neutral buffered 10% formalin, paraffin embedded, and sectioned on a microtome at 5 µm thickness. Slides were deparaffinized in a graded ethanol series, washed in water and boiled in antigen retrieval solution (10 mM sodium citrate, pH 6.0) prior to staining. The sources and dilutions of antibodies are: anti-cytokeratin 8 (1/200) (Developmental Studies Hybridoma Bank, Ames, IA), anti-estrogen receptor alpha (1/500) (Santa Cruz, sc-543), anti-progesterone receptor (1/500) (Santa Cruz, sc-538), anti-adaptin-alpha (1/800) (BD Transduction Lab, 610501), anti-clathrin heavy chain (1/500) (BD Transduction Lab, 610499), anti-NPT2b (1/500) (Alpha Diagnostics Int.), and anti-PMCA2 (1/500) (ThermoFisher Scientific). Other primary antibodies used for histology were the same as those used in immunoblotting. The dilutions for immunofluorescence microscopy were: anti-cleaved caspase-3 (1/300), anti-Dab2 (1/1,000), and anti-E-cadherin (1/1,000). Species-specific secondary antibodies were conjugated with the appropriate Alexa fluorochrome for simultaneous imaging of multiple antigens. DAPI (4′-6-diamidino-2-phenylindole) was used as a generic counterstain and applied in the terminal washing stages of the procedure. For immunohistochemistry, the secondary antibodies were HRP-conjugated (Vector Laboratories, CA, USA) and were detected by a DAB Peroxidase Substrate Kit (Vector Laboratories, CA, USA) followed by a hematoxylin counterstain.

### Laser scanning confocal microscopy

All confocal imaging was performed with an inverted Zeiss LSM510/uv Axiovert 200 M, laser scanning confocal microscope operated by Zeiss LSM software. Images were acquired with three sequential scan tracks. Objectives used included Plan-Apochromat (x63, 1.4 N/A) and Plan-Neofluar (x40, 1.3 N/A). Where necessary, images were contrast adjusted using Adobe Photoshop.

### Ultrastructural evaluation by transmission electron microscopy

Tissues were fixed in a two-step procedure: initially by immersion in 2% glutaraldehyde, 100 mM sucrose, 0.5 M phosphate buffer, pH 7.3; followed by treatment with 2% osmium tetroxide in 1 M phosphate buffer. After a graded dehydration, the tissues were embedded by infiltration with Epon/Araldite resin (Electron Microscopy Sciences, Fort Washington, PA) and an overnight polymerization at 64°C. Thin sections were cut using a Leica Ultracut R ultramicrotome with a Diatome diamond knife, then mounted on copper grids and contrasted with 4% uranyl acetate and 0.25% lead citrate. The sections were examined *in vacuo* with electrons accelerated at 60 kV and focused using the magnetic lens of a Philips CM10 transmission electron microscope.

## Results

### Induction of Dab2 expression in mammary glands during pregnancy and lactation

**Figure 1:**
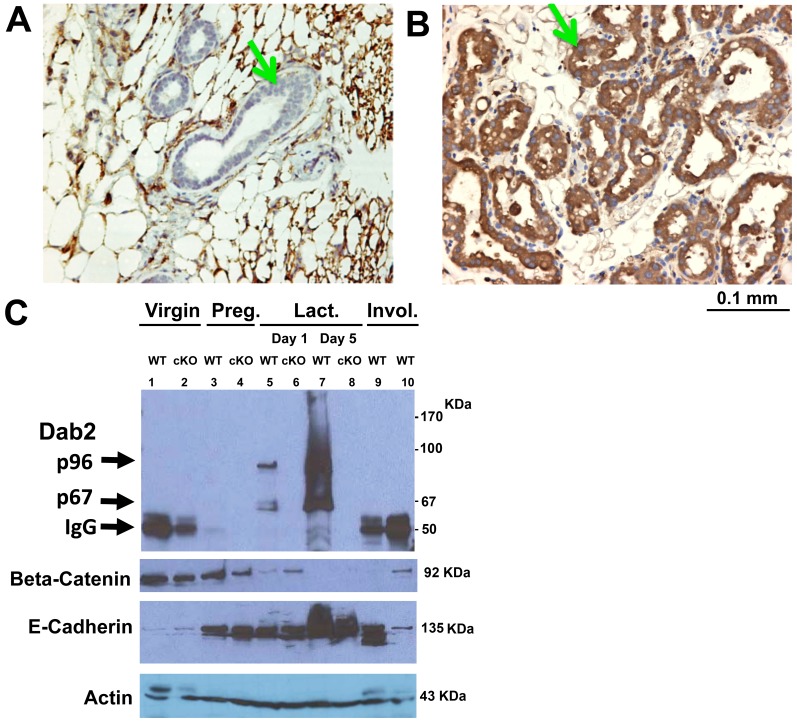
Induction of Dab2 by pregnancy and lactation in mammary glands. Mammary glands from virgin (**A**) and lactating (**B**) wildtype mice were harvested and used for histological analysis. Representative images of Dab2 immunostaining are shown. Arrows indicate the mammary epithelial cells. We also noted the positive staining of mammary adipocytes, which we have been confirmed to express Dab2 protein. (**C**) The induction of Dab2 proteins was confirmed by Western blot. Mammary glands were dissected and harvested from virgin, pregnant (Preg.) (12 days), and day 1 and day 5 of lactating (Lact.), and post-lactating/involuting (Invol.) wildtype (WT) mice. The post-lactation/involution samples were harvested from mothers that were weaned naturally. Specifically, the mice lactated for 3 weeks prior to separation from their pups for 1 (lane 9) or 3 (lane 10) days. Mammary tissues from conditional Dab2 knockout mice (*dab2* (f/df):Meox2-Cre) (cKO) were used as controls for antibody specificity. The tissues were lysed in SDS buffer, and equal protein from each sample was used for electrophoresis and Western blot analysis of Dab2, beta-catenin, and E-cadherin. Arrows indicate Dab2 p96 and p67 isoforms and a band that is presumably IgG heavy chain.

In a previous study of Dab2 using mouse models (Ref 56, and in more detail below), we observed that its expression in mammary glands varied greatly in different physiological stages. In virgin mice, the Dab2 protein was essentially undetectable in mammary epithelial cells by immunohistochemistry (**Figure 1A**, arrow), while Dab2 staining was robust and uniform in all mammary epithelial cells (arrow) of the mammary glands during lactation (**Figure 1B**, arrowhead). The induction of Dab2 protein isoforms in mammary glands was verified by Western blot analysis of tissue lysates (**Figure 1C**). While Dab2 was undetectable in virgin mammary glands, a low level appeared during pregnancy (15.5 days), and several isoforms, including p96 and p67, were massively induced upon lactation. In the involuting mammary glands, Dab2 proteins were lost (**Figure 1C**). Mammary tissue extracts from Dab2 conditional knockout mice were used to distinguish Dab2 isoforms from non-specific signals in the Western blots. Since mammary tissues contain multiple cell types, such as stromal, adipocytes, and immune cells, in addition to epithelial cells, we assayed E-cadherin as an indicator of epithelial content (**Figure 1C**). Beta-catenin was also probed, and it inversely correlated with E-cadherin levels. Based on equal protein loading and an equivalent beta-actin signal, the fraction of mammary epithelial cells was low in virgin, increased and remained similar in pregnant and day 1 lactating, and was highest in day 5 lactating mice (**Figure 1C**). In comparison, Dab2 proteins were not hormonally regulated in kidney epithelial or ovarian surface epithelial cells, suggesting a mammary-specific transcription co-factor(s) is required for regulation of Dab2 expression during pregnancy and lactation. The induction of Dab2 expression has been confirmed in multiple experiments using both Western blot, immunofluorescence microscopy, and immunohistochemistry, and these results indicate that in mammary glands, Dab2 expression commences in epithelia following pregnancy, reaches maximum during lactation, and wanes upon involution.

### Induction of Dab2 protein in mammary epithelial cells by reproductive hormones

**Figure 2:**
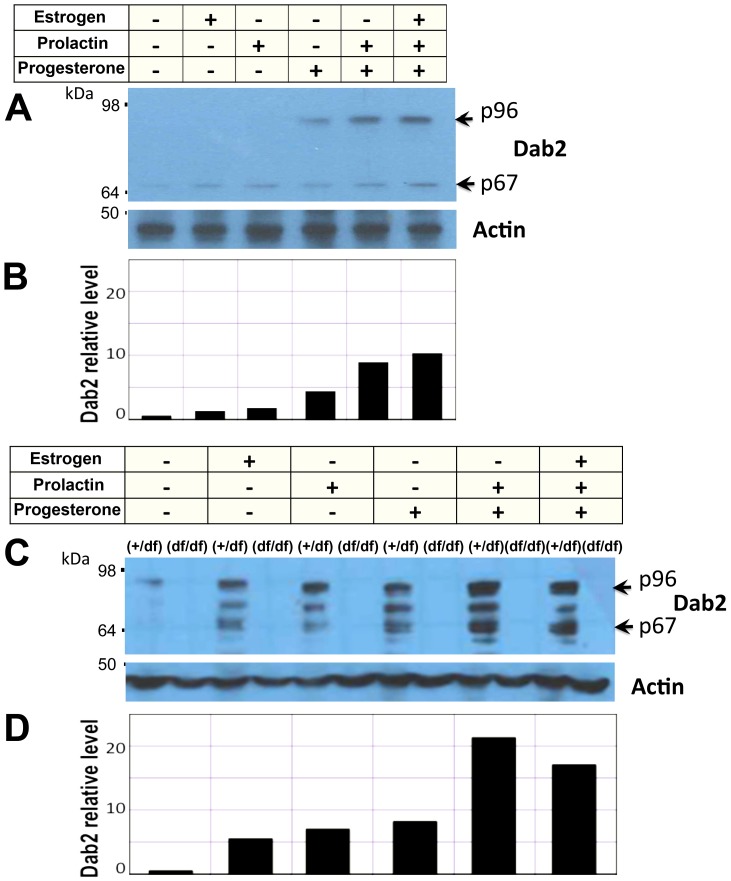
Hormonal induction of Dab2 expression in primary mammary epithelial cells. Mammary epithelial cells were prepared from virgin wildtype control (fl/+) and Dab2 conditional knockout (df/df) mice inheriting Meox2-Cre. The cells were treated with 17 beta estradiol (1 nM), progesterone (1 µM), and prolactin (1 nM, or 50 ng/ml), individually or in combination for 4 days in culture. (**A**) Dab2 and beta-actin from primary mammary epithelial cells of virgin mice were analyzed by Western blot. (**B**) The signals of Dab2 proteins from the Western blot were quantified using NIH Image J software using beta-actin as normalization control. Relative values were plotted with the value of untreated cells defined as 1.0. (**C**) Dab2 and beta-actin from primary mammary epithelial cells isolated from pregnant mice were analyzed by Western blot. Dab2 protein was absent in cells from the conditional knockout mice. (**D**) The relative intensity of Dab2 proteins on Western blots was quantified using NIH Image J software, using beta-actin for normalization.

Because Dab2 expression coincides with lactation, we speculated that Dab2 levels in mammary epithelial cells are regulated by reproductive hormones during lactogenic differentiation of mammary epithelial cells. We tested this possibility using primary mouse mammary epithelial cell cultures (**Figure 2**). In mammary epithelial cells isolated from mammary glands of virgin mice, progesterone but not estrogen or prolactin induced an about 4 folds increase in Dab2 proteins (**Figure 2A, B**). Progesterone and prolactin were synergistic in a higher induction to about 10 folds (**Figure 2A, B**). The mammary epithelial cell were isolated from expanded mammary glands of pregnant mice in order to produce sufficient number of cells for additional experiments, and the preparations were found to be more than 90% cytokeratin-positive. In these cultured cells, we found that Dab2 was regulated by physiological levels of estrogen, progesterone, and prolactin, while the combination of progesterone and prolactin was most potent in inducing Dab2 expression (**Figure 2C**). Several likely Dab2 isoforms, including the p96 and p67, were induced following exposure to hormones for 4 days. Mammary epithelial cells isolated from Dab2 knockout mice (more detail below) were used as controls for the specificity of Dab2 proteins in Western blot. The maximal induction of Dab2 proteins by prolactin plus progesterone was estimated to be 22-fold (**Figure 2D**), and the increase was similar in three repeat experiments.

### Mammary glands in mosaic Dab2 conditional knockout mice

**Figure 3:**
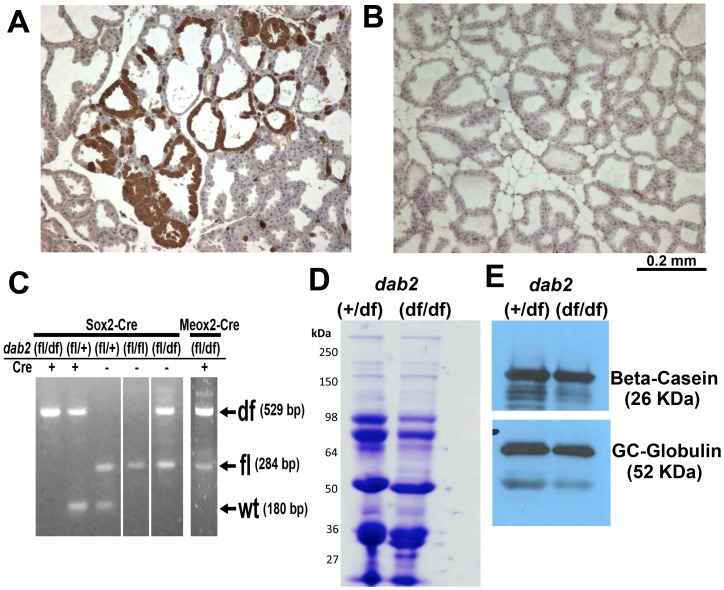
Normal lactation in mammary glands from *dab2* mosaic knockout mice. (**A**) Mammary glands from 4-month old, 5-day lactating mice of *dab2* (f/df):Meox2-Cre genotype show mosaic Dab2 immunostaining. (**B**) Dab2 was absent in more than 99% of mammary epithelial cells from mice of *dab2* (f/df):Sox2-Cre genotype. (**C**) DNA extracted from tail tissues was used to genotype flox (f) and delta flox (df) alleles of the *dab2* gene in *dab2* (+/df): Sox2-Cre, *dab2* (f/df):Meox2-Cre, and *dab2* (f/df):Sox2-Cre. (**D**) Milk was harvested from *dab2* heterozygous (*dab2* (+/df):Sox2-Cre) and knockout (*dab2* (f/df):Sox2-Cre) mice. Total proteins were resolved by SDS-PAGE and stained with Coomassie blue. (**E**) Specific proteins, beta-casein and GC-globin (vitamin D binding protein), were analyzed by immunoblot.

To bypass the requirement of Dab2 in embryonic development [11], [32] and to study the roles of Dab2 in mammary glands and adult tissues, we developed a Cre-lox conditional Dab2 knockout mouse model [56]. Using Meox2-Cre [57] or Sox2-Cre [58] to spare extraembryonic tissues but to delete *dab2* gene in embryonic proper in a mosaic fashion, we found that the resulting *dab2* knockout mice were grossly normal [56]. By immunostaining, Dab2 deletion in adult mammary glands was estimated to be about 95% in *dab2* (f/df): Meox2-Cre with Dab2-positive epithelial cells clustered focally (**Figure 3A**). However, the deletion was highly efficient in *dab2* (f/df): Sox2-Cre mammary glands and essentially no Dab2 positive cells were detected (**Figure 3B**). The degree of deletion agreed with PCR genotyping of tail DNA (**Figure 3C**).

When comparing virgin females of knockout, heterozygous, and wildtype, we did not recognize consistent or significant differences in the morphology of mammary glands between the genotypes of either Meox2-Cre- or Sox2-Cre-mediated deletion. The mammary glands of the conditional knockout mice also underwent normal branching morphogenesis as did the wildtype and heterozygous controls during pregnancy and lactation. We performed both whole mount and histological analysis of mammary glands from pregnant mice and analyzed branching morphogenesis and histology to observe gross epithelial structural alterations in a series of time courses; however, no consistent differences were detected.

Next, we investigated if there was a functional deficiency in the Dab2 mosaic mammary glands. The rationale was that Dab2 acts in endocytic trafficking and may influence uptake or secretion of proteins, as reported in kidneys [32] and in transport of vitamin D-binding proteins in human mammary cells [60]. We compared milk collected from Dab2 proficient and deficient mice. The major protein components of milk were identified by Coomassie staining of SDS-PAGE gels (**Figure 3D**), and specific proteins beta-casein and Vitamin D binding protein (GC-globin) were detected by Western blot (**Figure 3E**). Any quantitative and qualitative differences in milk content were subtle between knockouts and controls. Furthermore, Dab2-deficient mothers were capable of normal nursing. Thus, loss of Dab2 protein has minimal impact on mammary milk production and nurturing litters.

**Figure 4:**
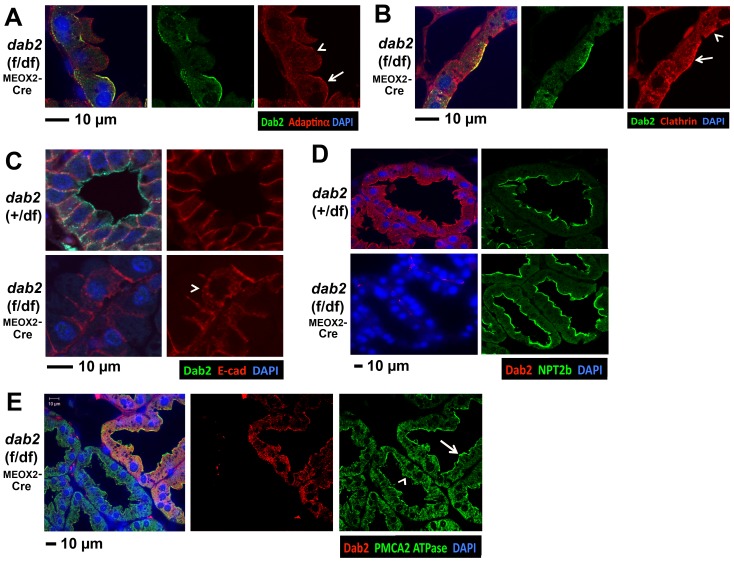
Mild effects of Dab2 deletion on endocytic components and polarity markers in mammary epithelia. Mammary glands from lactating (5 days) mice of *dab2* heterozygous controls and mosaic knockouts (*dab2* (f/df):Meox2-Cre) were analyzed by confocal immunofluorescence microscopy for several endocytosis and polarity markers. Where possible, markers were compared between Dab2-positive and Dab2-negative cells in the same section of the mosaic *dab2*-deleted tissues. (**A**) Adaptin alpha and Dab2 double staining of mammary glands from a mosaic *dab2*-deleted mouse, comparing Dab2-positive (arrow) and negative (arrowhead) cells; (**B**) Clathrin and Dab2 co-staining of mammary glands from a mosaic *dab2*-deleted mouse, comparing Dab2-positive (arrow) and negative (arrowhead) cells; (**C**) Dab2 and E-cadherin staining: E-cadherin is localized more in the cytoplasm in Dab2-deleted cells (arrowhead); (**D**) NPT2b, a sodium-phosphate cotransporter; and (**E**) PMCA2 ATPase, a calcium pump, in Dab2-positive (arrow) and negative (arrowhead) cells. The results are the representation of multiple slides from 3 control (wildtype and heterozygous) and 3 *dab2* conditional knockout mice.

We further examined the impact of Dab2 deletion on the distribution of other endocytic components. In lactating mammary glands with a mosaic *dab2* deletion, we compared adaptin-alpha (**Figure 4A**) and clathrin (**Figure 4B**) in adjacent Dab2-positive and negative epithelial cells in the same section, and noticed slight but consistent differences in the distribution of these endocytic proteins. Dab2-positive cells had a more intense apical localization of adaptin-alpha and clathrin (**Figure 4A and B**, arrow) than Dab2-negative cells (**Figure 4A and B**, arrowhead). Since Dab2 has a role in maintaining polarity and epithelial organization of extraembryonic endoderm [12], we further examined E-cadherin and other polarity markers in mammary epithelial cells. E-cadherin showed an overwhelmingly basolateral distribution in the Dab2-positive mammary epithelial cells; however the staining was more cytoplasmic and punctated in Dab2-negative cells (**Figure 4C**, arrowhead). No obvious changes were detected in the distribution of the apical marker, sodium/phosphate co-transporter NPT2b [61] (**Figure 4D**). Nevertheless, a loss or reduced apical distribution of the calcium pump PMCA2 [62] was evident in the Dab2-negative (**Figure 4E**, arrowhead) compared to adjacent Dab2-positive cells (**Figure 4E**, arrow). We have consistently detected the genotype-dependent changes in multiple slides from 3 controls (both wildtype and heterozygous) and 3 conditional deletions. Additionally, when available, we used slides containing regions showing Dab2-positive and negative adjacent cells for analysis to demonstrate a change depending on Dab2 expression.

Thus, Dab2 loss alters the polarized distribution of certain endocytic, cell adhesion, and membrane proteins such as E-cadherin, PMCA2, adaptin-alpha, and clathrin, but does not impact the gross structure of mammary epithelium or its function in lactation.

### Delayed mammary involution in Dab2 conditional knockout mice

**Figure 5:**
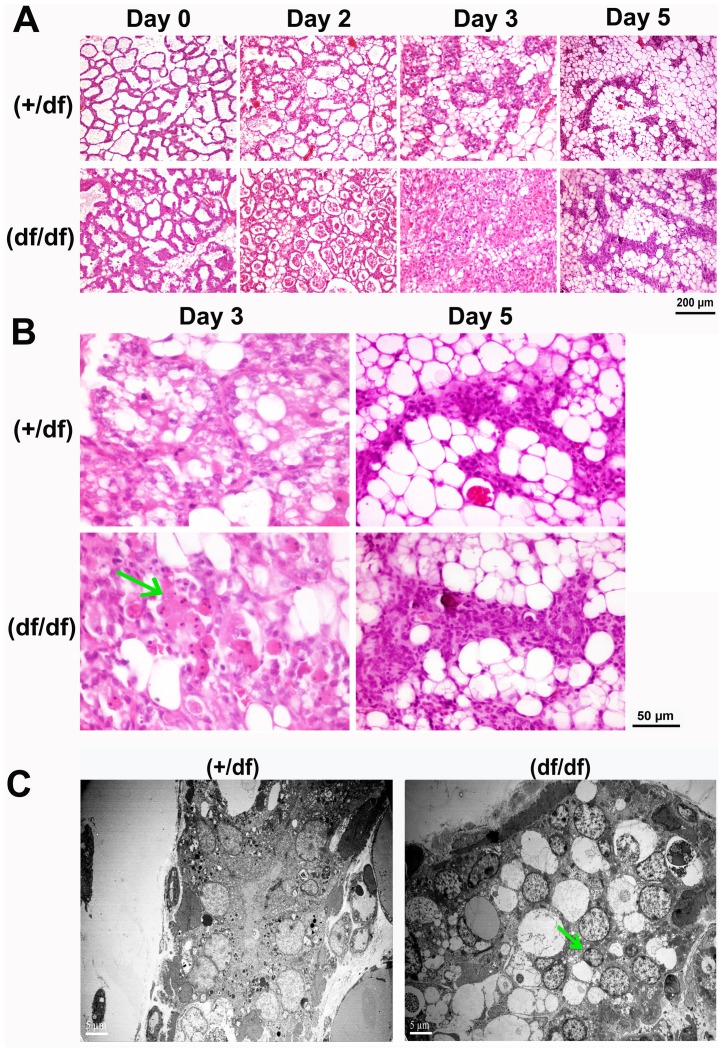
Dab2 deficient mammary glands have delayed involution. A control (*dab2* heterozygous) and *dab2* knockout (*dab2* (f/df):Sox2-Cre) group of six 6-month old mice were mated, became pregnant, gave birth, and nursed an equal number (six) of pups per mouse. At day 12 of lactation, the mice underwent forced involution by removal of the pups. Mammary glands were harvested accordingly for histological analysis. Consistent results were obtained in two independent experiments using deletion by either Meox2-Cre or Sox2-Cre. (**A**) Representative H&E images show the morphology of the mammary glands. (**B**) Images of higher magnification are shown for day 3 and 5 of involution. Accumulating apoptotic cells in the lumen are indicated by a green arrow. (**C**) Mammary tissues of heterozygous and *dab2* knockout at day 3 of forced involution were analyzed by transmission electron microscopy. An arrow indicates the presence of cells in the interior of the *dab2* knockout mammary lumens.

Despite the induction of Dab2 in wildtype mammary glands, the Dab2-deficient females progressed through pregnancy, lactation, and nursing without any obvious problems. However, we consistently observed that the kinetics of mammary regression were retarded in the Dab2-deficient mammary glands, in which cells with condensed nuclei persisted and cell clearance was delayed (**Figure 5**). Lactating female mice were separated from their pups 12 days after birth to initiate forced mammary involution, and mammary tissues were analyzed. Accumulation of cells and debris was evidenced in the alveolar lumens of Dab2-deficient mammary glands at day 2 of involution, compared to controls (**Figure 5A**). In the heterozygous control group, epithelial alveoli regressed greatly by day 3, while adipose cells repopulated the glands. In contrast, Dab2-deficient mammary tissues were still composed mostly of epithelial components at this stage, and few adipocytes were present. Images at higher magnification showed that the lumens harbored a large number of rounded cells with condensed nuclei at day 3 of involution in the Dab2-deficient mammary glands (**Figure 5B**, arrow). Such cells were present but scarce in control mammary glands. However, by day 5, the differences became minimal, and Dab2-deficiency seemed only to delay but not incapacitate epithelial regression in mammary involution (Figure 5A, B). We have repeatedly detected the delayed mammary involution in multiple (at least 5) independent experiments using groups (3–6 mice in each groups) of control and Dab2-null mice over a period of 2 years. Thus, the impact of Dab2 in mammary involution, although transient, is robust and consistent.

We further used electron microscopy to examine the day-3 involuting mammary glands for differences between control and Dab2-deficient mice (**Figure 5C**). In the Dab2-deficient mammary glands, an increased number of vacuoles and nuclei were observed in the interior of the lumens (**Figure 5C**, arrow).

Since the most noticeable differences between control and Dab2-deficient mammary glands occurred on day 3 of involution, we further characterized the phenotypes at this stage using several markers. First, we examined the presence of macrophages because this cell type has a high level of Dab2 [3] and its absence may reduce the ability of these cells to engulf and clear dead cells and debris. However, no significant differences were observed: similar numbers of F4/80-positive macrophages were present in both control and Dab2-deficient tissues, and mostly located outside rather than within the alveolar lumens.

**Figure 6:**
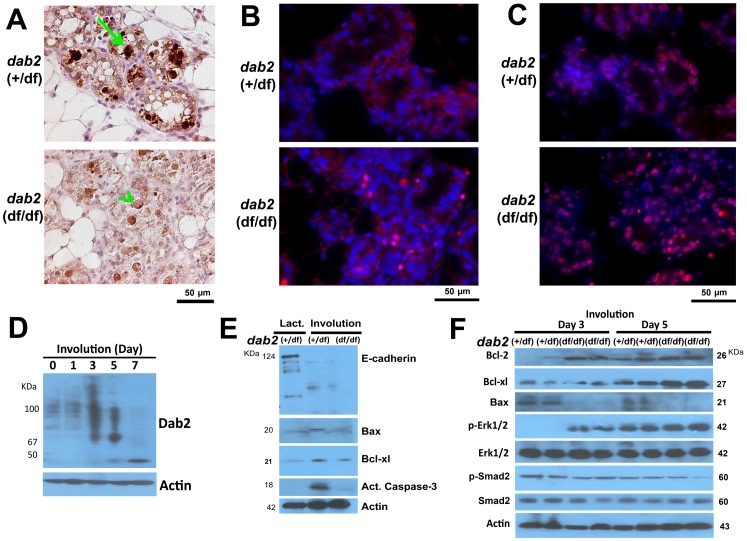
Delayed apoptosis of Dab2-deficient mammary epithelial cells during involution. (**A**) The day 3 involuting mammary glands from control (*dab2* heterozygous) and *dab2* knockout (*dab2* (f/df):Sox2-Cre) mice were analyzed. Apoptotic cell death in situ indicated by immunostaining for activated caspase-3, comparing wildtype (arrow) and Dab2 deficient cells (arrowhead). (**B**) A representative immunofluorescence microscopy staining for phospho-Erk1/2 in Dab2 heterozygous and knockout mammary glands on day 3 of involution. Phospho-Erk1/2 (red) overlaying on DAPI (blue) stainings are shown. (**C**) Immunofluorescence microscopy for Bcl-2 (red) and DAPI (blue) staining of day 3 involuting mammary glands. (**D**) Dab2 expression was determined by Western blot of tissue extracts of involuting mammary glands from *dab2* heterozygous mice at 0, 1, 3, 5, and 7 days following forced involution. (**E**) Western blot analysis of E-cadherin, Bax, Bcl-xl, and activated caspase-3. Mammary protein extracts from heterozygous lactating (day 12) and forced involuting (day 3) mice were analyzed. (**F**) Western blot analysis of Bcl-2, Bcl-xl, Bax, phospho-Smad2, total Smad2, phospho-Erk1/2, and total Erk1/2 in protein lysates extracted from mammary glands following 3 and 5 days of forced involution. Two independent samples (duplicate) of each genotype from different mice are shown in the blot.

In control day-3 involuting mammary glands, intensive focal staining of cleaved caspase-3 indicated active apoptosis (**Figure 6A**, arrow); however in comparison, the staining of many Dab2-deficient mammary epithelial cells appeared lighter and diffuse, and few clear caspase-3-positive cells were seen (**Figure 6A**, arrowhead). The Dab2 null mammary glands showed an increased activation of Erk1/2 since that 16% of the cells were phospho-Erk1/2 positive in nuclei; in contrast, few (<1%) cells were positive for nuclear phospho-Erk1/2 in control mammary glands (**Figure 6B**). Consistently with an increased Erk1/2 activation, 82% of the day-3 involuting Dab2 null mammary cells were positive for Bcl-2, compared to 26% in control (*dab2* heterozygous) cells (**Figure 6C**).

In Western blot analysis of protein extracts from involuting mammary glands, we found that at 3 day, Dab2 protein level increased and presented as a higher molecular weight smear (**Figure 6D**). This smear might be modified proteins (such as ubiquitin conjugated), and we are currently investigating further. Dab2 p96 and p67 proteins were absent by day 7 of involution. Western blots also showed the reduction of E-cadherin in both Dab2-positive and negative mammary glands, and the pro-apoptotic proteins Bax and activated caspase-3 were greater in controls than the Dab2-deficient tissues (**Figure 6** **E** and **F**). Levels of the pro-survival proteins, particularly Bcl-2, were significantly elevated in Dab2 conditional knockout mammary glands compared to heterozygous controls (**Figure 6F**). Notably, we found that the phosphorylation and activation of Erk1/2, a pro-survival signal, were augmented on day 3 of involution in Dab2-deficient mammary glands (**Figure 6F**). On day 5, the differences in Erk1/2 activation and expression of apoptotic regulators were diminished between Dab2-proficient and deficient mammary glands (**Figure 6F**). No significant difference in phospho-Smad2 was observed between Dab2-posoitive and deficient tissues (**Figure 6F**).

Thus, a consequence of *dab2* deletion in mammary glands is the unsuppressed Erk activation, increased pro-survival mediators (such as Bcl-2), lessened apoptotic activation (such as Bax and activated caspase-3), and ultimately delayed cell death and clearance.

### Growth and signaling of *dab2* knockout mammary epithelial cells in vitro

**Figure 7:**
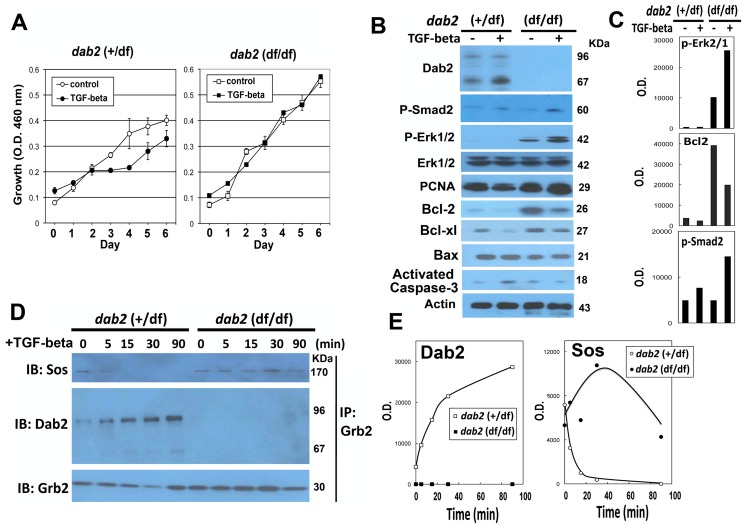
Dab2 modulates TGF-beta-stimulated growth regulation and signaling in primary mammary epithelial cells. Mammary epithelial cells were prepared from pregnant control (+/df) and *dab2* conditional knockout (df/df) mice inheriting Sox2-Cre. The cells were treated with or without TGF-beta (10 ng/ml). (**A**) Growth of the cells was determined by WST assay over a 6-day period. Student-t test indicated that the difference in cell growth was statistically significant for at day 3 to 6 for *dab2* (+/df) (p<0.005) but not *dab2* (df/df) cells. (**B**) Protein lysates prepared from the primary cultures were analyzed by Western blots for Dab2, phospho-Smad2, E-cadherin, N-cadherin, phospho-Erk1/2, total Erk1/2, PCNA, Bcl-2, Bcl-xl, Bax, activated caspase-3, and beta-actin. (**C**) The relative protein level was quantified from the Western blots using NIH Image J software, and the values of the optical density (O.D.) critical markers (p-Smad2, p = Erk1/2, and Bcl-2) were compared. (**D**) Co-immunoprecipitation was performed to determine the association between Grb2 and Sos or Dab2. The primary cells were stimulated by TGF-beta for a time course of 0, 5, 15, 30, and 90 min. At each time point, the monolayer was washed with ice-cold PBS, lysed, and the post-nuclear supernatants were used for immunoprecipitation with antibodies against Grb2. The eluted proteins from the immunoprecipitation were separated by SDS-PAGE, and immunoprobed for Sos, Dab2, and Grb2. The immunoprecipitation experiments were performed twice and similar results were obtained. (**E**) Relative Dab2 and Sos protein amounts in the Western blot were estimated using NIH Image J program and the O.D. values were plotted.

Since TGF-beta signaling is known to be critical in mammary involution [48], [63], [64] and several reports suggest a role of Dab2 in the regulation of this pathway. We investigated TGF-beta signaling and growth control in primary mammary epithelial cells isolated from *dab2* knockout and control mice. Unlike involution in vivo, TGF-beta failed to induce significant cell death in cultures of primary mammary epithelial cells. Nevertheless, upon TGF-beta exposure, the wildtype mammary epithelial cells showed a reduced cell proliferation (**Figure 7A**). However, Dab2-deficient cells exhibited an unsuppressed proliferation and were refractory to TGF-beta induced growth inhibition (**Figure 7A**).

Dab2 deficiency did not eliminate canonical TGF-beta signaling, indicated by the phosphorylation and activation of Smad2 (**Figure 7B**), but led to a higher basal and TGF-beta-stimulated Erk1/2 activation (**Figure 7B, C**). Additionally, we observed a slight increased amount of PCNA (an indicator of cell proliferation), and an increased Bcl-2 level in Dab2-deficient compared to Dab2-proficient cells (**Figure 7B**). Bax and activated caspase-3 levels were not significantly altered (**Figure 7B**), consistent with the lack of extensive TGF-beta induced apoptosis in the cultured cells. The TGF-beta signaling experiments were performed 5 times, and the results were entirely consistent. In summary, TGF-beta suppressed growth of wildtype mammary epithelial cells in vitro. However, the suppression was abolished in Dab2-deficient cells, accompanied by an increased Erk1/2 activation (**Figure 7C**).

We further tested the molecular mechanism for the increased phospho-Erk1/2 in the absence of Dab2. Several previous studies have suggested that Dab2 binds Grb2, competing with Sos and thus suppressing the Ras/MAPK pathway [3], [23]. In primary mammary epithelial cells, co-immunoprecipitation was used to assay the competitive association between Grb2 and Sos or Dab2 (**Figure 7D**). In Dab2-positive control cells, TGF-beta stimulation led to a progressively increased association between Grb2 and Dab2 and a declining binding of Grb2 with Sos. In the absence of Dab2, persistent Grb2 and Sos interaction was maintained as shown by immuno-coprecipitation and Western blot (**Figure 7D, E**). Thus, the deletion of Dab2 led to an increased Grb2-Sos association and an unsuppressed TGF-beta-stimulated MAPK activation in mammary epithelial cells.

## Discussion

**Figure 8:**
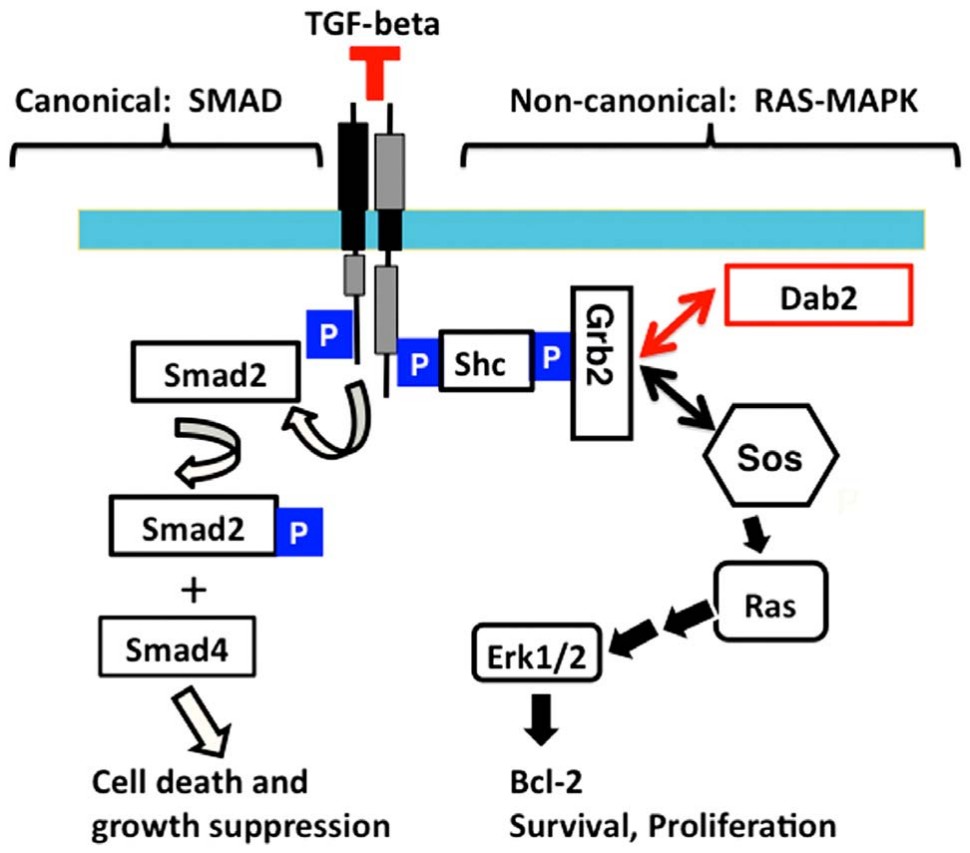
Schematic illustration of a working model for Dab2 in modulation of TGF-beta pathway. Canonical TGF-beta signaling pathway consists of Smad phosphorylation and mediation of transcriptional regulation, leading to cell death and growth suppression, representing a tumor suppressor activity. The non-canonical route includes the activation of Ras/MAPK pathway as a result of phosphorylation and binding of TGF-beta receptor to Shc and consequently recruitment of Grb2 and Sos. TGF-beta-stimulated activation of Ras/MAPK pathway induces expression of genes involved in cell survival and growth. Dab2 modulates TGF-beta signaling by sequestering Grb2 from Sos, resulting in a reduction of Ras/MAPK activation yet allowing Smad-mediated gene transcription.

The current study reports the induction of Dab2 expression and the phenotype of mammary glands in Dab2 conditional knockout mice. Dab2 deficiency delays epithelial cell death and clearance during mammary involution. We have provided data to suggest a working model whereby Dab2 expression is induced during lactation to modulate TGF-beta signaling by suppressing TGF-beta-stimulated MAPK activation. Dab2 retards MAPK activation by competing with Sos for binding to Grb2 and thus ultimately suppresses the signaling pathway (**Figure 8**).

The current finding that estrogen, progesterone, and prolactin induce expression of Dab2, a growth and tumor suppressor, may represent a feedback mechanism for growth control. It is well accepted that increased estrogen exposure, such as early menarche and late menopause, is a potential risk factor for breast cancer [42], [65]. On the other hand, hormonal exposure during pregnancy and lactation at an early age offers some protection [65]–[68]. This epidemiological observation generates great interest, since breast cancer has few practical preventive measures. One idea is to use pregnancy or lactation hormones for short-term treatment in young women as a potential prophylactic strategy for reducing breast cancer risk [67], [69], [70]. Another contemplation is to mimic the potential protective biology of pregnancy by using human chorionic gonadotropin [71]. It is reasoned that pregnancy and lactation enforce the differentiation of mammary epithelial cells, and thus reduce the presence of undifferentiated or stem-like precursor cells that have a greater potential for neoplastic transformation [72]. Furthermore, post-lactational involution may purge pre-neoplastic cells, but dysregulation of the process could facilitate tumor formation [68] Our finding suggests that Dab2 may be one of the genes involved in providing a protective effect for pregnancy against breast cancer risk.

Dab2 was found expressed widely and particularly high in kidney epithelial cells [4], [56]. The current findings of the induction of Dab2 expression in pregnancy/lactation and by estrogen, progesterone, and prolactin in cultured mammary epithelial cells are surprising, since Dab2 was not known to be hormonally regulated. In sequence analysis of the DAB2 promoter [73], [74], an estrogen responsive element, ggtca gaa tgacc (the conserved bases are underlined) [75], [76], was found at around 4 kb upstream of the transcriptional start site. The sequence is conserved at this site between mouse and human [73], [74]. Although we found that Dab2 expression was greatly stimulated by estrogen, progesterone, and prolactin in primary cultures of mammary epithelial cells isolated from pregnant mice, we did not observe a significant increased Dab2 expression in mammary glands following systemic delivery of estrogen or progesterone in vivo. Additionally, Dab2 was not inducible by estrogen and progesterone in primary mammary epithelial cells isolated from virgin mice, human breast epithelial cells, or estrogen-dependent breast cancer cells (such as MCF-7). We speculate that Dab2 hormonal induction requires priming of the mammary epithelial cells by additional factor(s) produced during pregnancy. However, the in vivo environment also prevents the complete induction of Dab2 by estrogen and progesterone during pregnancy, and Dab2 is only fully induced during lactation by the simultaneous presence of estrogen, progesterone, and prolactin.

**Figure 9:**
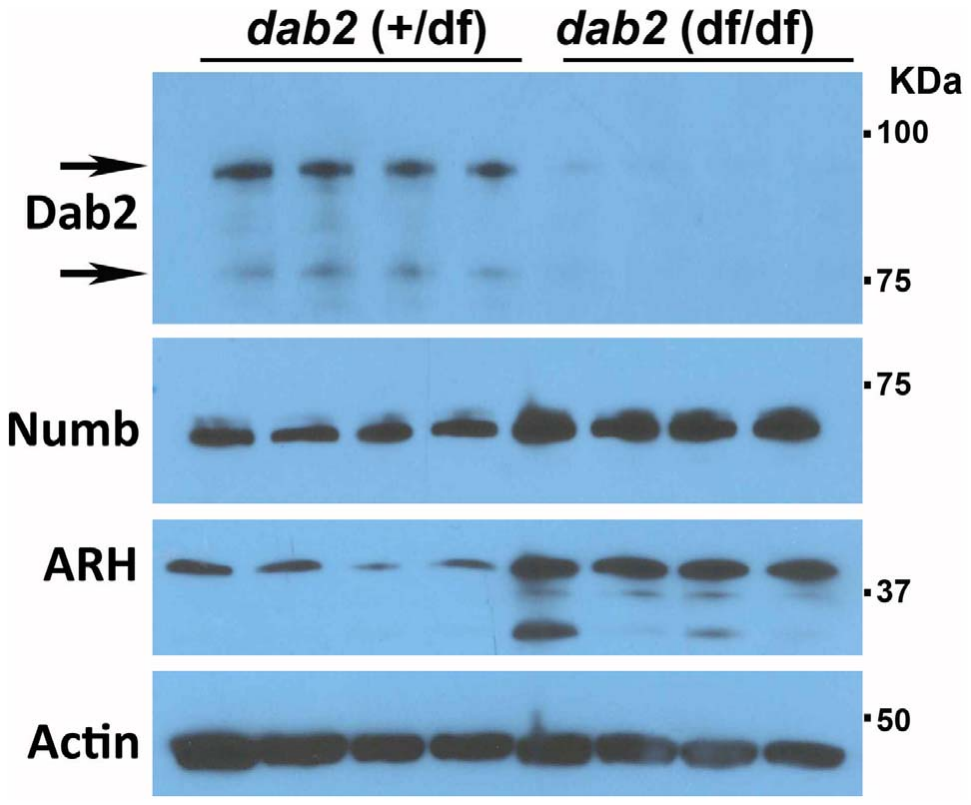
Compensatory expression of ARH and Numb in Dab2 null mammary epithelial cells. Dab2, ARH, and Numb proteins were analyzed by Western blotting of primary mammary epithelial cells isolated from control (*dab2* wildtype and heterozygous (*dab2* (+/df)) and conditional knockout (*dab2* (f/df); Meox2-Cre) 16.5-day pregnant mice. Based on quantitation using Image J and averaging the outputs, Numb increased 2.1-fold and ARH increased 4.3-fold in Dab2 null cells.

The endocytic function of Dab2 may provide manifold functions in mammary glands during lactation, such as nutrient uptake, milk production and secretion, cell growth, survival, and clearance of dead cells and debris. However, only subtle differences in mammary functions were observed between control and Dab2-deficient mice. The lack of more profound defects in Dab2 knockout mammary glands may due to the compensation by other PTB domain containing endocytic adaptors such as Numb and ARH. Indeed, we have found that Numb and ARH protein levels are increased in Dab2 knockout mammary gland epithelial cells (**Figure 9**). Previously, we have also observed a compensatory expression of Numb and ARH in Dab2-null mouse ES cells and embryos [56].

Mammary involution, the regression of mammary epithelia in a relative short time frame (4-5 days in mice), is a highly regulated process [46], [49]. Many proteins involved in cell death and survival, such as Bax, Bcl-2, and Akt, play important roles in involution [50], [53], [54], and the TGF-beta signaling pathway is known to be crucial [47]. The canonical pathway of TGF-beta signaling involves the phosphorylation of Smad family proteins, which then dimerize with their co-Smad partners and enter the nucleus to modulate transcription [77]. TGF-beta also activates non-canonical pathways including the Ras/MAPK cascade [78]–[83]. The mechanism is that TGF-beta receptor phosphorylates and associates with Shc directly, which then recruits Grb2-Sos complex to activate Ras [80]. The TGF-beta mediated Ras/MAPK activation counters its tumor suppressive signaling branch through Smad activation [84], [85], and Ras/MAPK activation induces survival and suppresses apoptotic genes [86], [87]. Earlier observations indicate that Dab2 is a direct binding partner of Grb2, competing with Sos, and thus can modulate Ras/MAPK pathway in certain circumstances [3], [23]. Our results suggest that the induction of Dab2 suppresses TGF-beta-induced Erk1/2 activation during mammary involution, which may explain the prolonged survival of Dab2-null mammary epithelial cells during involution because of the unsuppressed TGF-beta-induced Ras/MAPK activation.

Another possible mechanism for Dab2 in mammary involution is a role in macrophage-mediated clearance of epithelial cells. We did not observed a difference in macropahge density in the involuting glands, though it is thought that epithelial cell-directed efferocytosis is important [88]. Thus, it is possible that Dab2-null mammary epithelial cells are less efficient in cell clearance during mammary regression.

The participation of Dab2 in TGF-beta regulation was first suggested to mediate the receptor activation of Smad2/3 [25]. We did not detect any effect of Dab2 deletion on Smad2 activation; rather, Dab2 suppresses TGF-beta stimulated Erk1/2 activation. Thus, the results suggest that the induction of Dab2 in mammary epithelial cells leads to the unobstructed TGF-beta stimulated activation of Smad2/3, a growth suppressive signal, and suppression of TGF-beta stimulated activation Erk1/2, a growth-stimulating signal. Thus, a model is proposed that Dab2 suppresses TGF-beta-stimulated MAPK activation by competing with Sos to bind Grb2-Shc and thus reducing the degree of Ras/MAPK activation (**Figure 8**). Dab2 expression is often lost in cancers, including breast cancer [9], [10]. Thus, loss of Dab2 may account for the elimination of TGF-beta growth suppressive activity due to the unsuppressed Erk1/2 activity. Dab2 appears to be a factor determining the context dependence of TGF-beta signaling [89].

In sum, we report here that Dab2 expression is induced in mouse mammary glands during pregnancy and lactation. We conclude that Dab2 plays a role in strengthening epithelial organization and modulating TGF-beta signaling, and functions in enhancing apoptotic clearance of mammary epithelial cells during involution.
